# Television Time among Brazilian Adolescents: Correlated Factors are Different between Boys and Girls

**DOI:** 10.1155/2014/794539

**Published:** 2014-02-25

**Authors:** Diego Augusto Santos Silva, Mark Stephen Tremblay, Eliane Cristina de Andrade Gonçalves, Roberto Jerônimo dos Santos Silva

**Affiliations:** ^1^Federal University of Santa Catarina, Post-Graduate Program of Physical Education, Florianópolis, SC, Brazil; ^2^Campus Universitário Reitor João David Ferreira Lima, Centro de Desportos, Departamento de Educação Física, Bairro Trindade 88040-900, Florianópolis, SC, Brazil; ^3^Healthy Active Living and Obesity Research Group, Department of Pediatrics, University of Ottawa, CHEO Research Institute, 401 Smyth Road, Ottawa, ON, Canada K1H 8L1; ^4^Federal University of Sergipe, Physical Education Department, Aracaju, SE, Brazil

## Abstract

*Objective*. The aim of this study was to identify the prevalence of excess television time and verify correlated factors in adolescent males and females. *Methods*. This cross-sectional study included 2,105 adolescents aged from 13 to 18 years from the city of Aracaju, Northeastern Brazil. Television time was self-reported, corresponding to the time spent watching television in a typical week. Several correlates were examined including age, skin color, socioeconomic status, parent education, physical activity level, consumption of fruits and vegetables, smoking status, alcohol use, and sports team participation. *Results*. The prevalence excess television time (≥2 hours/day) in girls and boys was 70.9% and 66.2%, respectively. Girls with low socioeconomic status or inadequate consumption of fruits and vegetables were more likely to have excess television time. Among boys, those >16 years of age or with black skin color were more likely to have excess television time. *Conclusions*. Excess television time was observed in more than two-thirds of adolescents, being more evident in girls. Correlated factors differed according to sex. Efforts to reduce television time among Brazilian adolescents, and replace with more active pursuits, may yield desirable public health benefits.

## 1. Introduction

Research on sedentary behaviors has gained attention in the academic community and is the subject of public health messages for behavior modification [1–3]. Conceptually, sedentary behavior is a term used to describe activities performed in the sitting or reclining position (such as television viewing and computer use, travelling in a car, time sitting talking, reading, and talking on the phone), which show energy expenditure values close to resting values (<1.5 METs) [4, 5]. Sedentary behavior is a construct different from physical inactivity, with specific determinants and distinct implications for people's health [2]. Physical inactivity is the absence of physical activity, usually reflected as the proportion of time not engaged in physical activity of a predetermined intensity [3]. Importantly, it is possible to meet physical activity recommendations and still engage in high amounts of sedentary behavior [3]. A number of prospective epidemiologic studies have demonstrated that prolonged sedentary behavior promotes deleterious health effects that may be independent of the practice of physical activity [6].

Television time is one of the most investigated sedentary behaviors because most of the population has such electronics, especially in middle-income countries such as Brazil. Tremblay et al. [7] carried out a systematic review in order to establish the relationship between sedentary behavior and health outcomes in children and adolescents aged 5–17 years. The health outcomes examined included body composition, physical fitness, metabolic syndrome and cardiovascular risk factors, self-esteem, behavioral and social conduct, and academic performance. The authors demonstrated that young people with sedentary risk behavior were more prone to gain weight, to be overweight or obese, and to have poor performance on physical fitness tests, higher levels of blood pressure, total cholesterol and triglycerides, higher chances of showing depressive symptoms, low self-esteem, aggressiveness, inappropriate social behaviors, and poor academic performance [7]. These authors and others have noted that the vast majority of the literature available related to screen time exposure, and especially television viewing [7, 8]. Further investigation of factors related to sedentary behaviors in general, and television time specifically, may help identify the groups most prone to these behaviors.

Systematic reviews on the subject [6, 7, 9, 10] did not address sedentary behavior determinants stratified by sex. However, boys and girls have different habitual behaviors, resulting from both biological and cultural factors that influence the way they interact with the environment. Thus, it is believed that factors related to sedentary behaviours, like television viewing, differ according to sex.

The aim of this study was to identify the prevalence of excess television time (≥2 hours/day) and determine correlates of this behavior in adolescent males and females in Northeastern Brazil.

## 2. Methods

This cross-sectional epidemiological study was carried out in the metropolitan area of the city of Aracaju, Sergipe, Brazil. The state of Sergipe is the smallest state of Brazil and is located in the northeastern region with 75 counties and the best Human Development Index (HDI) in the region (0.742), with life expectancy of 70.3 years, infant mortality rate of 21 children among one thousand live births, and illiteracy rate among people aged 15 and older of 16.9% [11, 12]. The city of Aracaju is the capital of Sergipe and has an estimated population of 571,149 inhabitants, poverty incidence of 27.5%, Gini index of 0.47%, and HDI of 0.794 [11, 12]. HDI is a composite index that measures the average achievements of a given city in three basic dimensions of human development: (a) long and healthy life, as measured by life expectancy at birth, (b) knowledge, as measured by the adult literacy rate and the ratio of combined gross enrolment in primary, secondary, and higher schools, and (c) a decent standard of living, as measured by gross domestic product (GDP) per capita in purchasing power parity in US dollars [12]. The Gini index, which is expressed in percentage points, is a measure commonly used to calculate income distribution inequalities. It consists of a number between 0 and 100, where 0 corresponds to complete income equality and 100 corresponds to complete inequality [11, 12].

### 2.1. Population and Sample

The target population of this study consisted of 13,373 high school students from public schools of Aracaju and metropolitan area. High school in Brazil consists of three school grades (1st, 2nd, and 3rd).

The sample size was calculated to estimate the prevalence of different health outcomes investigated in the survey, considering a confidence level at 95%, prevalence for unknown outcomes of 50%, sampling error of 3%, estimated design effect of 2.0 (due to cluster sampling), and percentage of losses estimated at 10%. Based on these parameters, sample size of 2,174 adolescents was obtained.

For association tests, considering an estimated outcome prevalence of 50%, power of 80%, and confidence level of 95%, this sample size would allow detecting a statistically significant odds ratio of up to 1.4 as risk factor and up to 0.6 as protection for females and up to 1.7 as risk factor and up to 0.5 as protection for males.

The sampling design included state public schools and was determined in two stages with school and then class as the sample units. All high schools of Aracaju and metropolitan area with enrollment exceeding 350 students were eligible for inclusion in the study. In the second stage, the density of classes in selected schools was considered as a criterion to draw those in which the questionnaires would be applied. All students in the selected classes were invited to participate in the study.

### 2.2. Inclusion and Exclusion Criteria

To participate in the study, the student needed to be enrolled in participating schools, be 13 to 18 years of age, and not have any condition that prevented him/her from understanding and completing questionnaire (e.g., blindness and deafness). Adolescents who declined to participate in the study and those whose parents/guardians did not provide consent were considered refusals.

### 2.3. Ethical Aspects

The study was approved by the Ethics Committee on Human Research of the Federal University of Sergipe, Brazil. All participants in the survey provided signed informed consent by parents (if under 18 years of age), or by themselves (≥18 years).

### 2.4. Data Collection

Data collection was held in the second half of 2011. The team of evaluators was composed of physical education graduation students who attended previous training for standardization of data collection procedures. The study instrument was a structured questionnaire designed in Portuguese. Data were collected at the school through self-completed questionnaire. The participants were assisted as required by the evaluators.

### 2.5. Dependent Variable

The questionnaire included a question on whether the adolescent watched television (TV). The translated question was (1) “How much time do you spend watching TV?” (in typical week). Participants who reported ≥2 hours per day watching TV were considered to have excess TV time. The Canadian sedentary behaviour guidelines for children and youth [3], the American Academy of Pediatrics [13], and other experts recommend that adolescents limit television and other screen time to no more than 2 hours per day.

### 2.6. Independent Variables

Sociodemographic variables included age in years (categorized into ≤16 years and 17-18 years); self-reported skin color, collected based on the categories of the Brazilian Institute of Geography and Statistics [14] and ranked as “white,” “lighter skinned black,” and “dark skinned black” (the results of those who reported as having yellow or indigenous skin color altogether accounted for less than 10% of the sample and therefore are not shown in tables but were included in the association analyses); paternal and maternal schooling (≤8 years and >8 years); and economic level that categorizes participants into economic classes according to family purchasing power [15]. The five economic classes “A,” “B,” “C,” “D,” and “E” represent decreasing order of purchasing power. Subjects from classes “A” and “B” were classified as “high” economic level, those from class “C” as “intermediate” economic level, and those from classes “D” and “E” as “low” economic level.

Physical activity level, consumption of fruits and vegetables, smoking status, alcohol use, and participation in team sports were assessed by the Brazilian version of the questionnaire used in the Youth Risk Behavior Surveillance System (YRBSS) used in the United States. The YRBSS questionnaire was translated into Portuguese and validated for Brazil, and the behaviors investigated in this study showed Kappa agreement index from moderate to high [16].

Physical activity level was assessed by the following question: during the past 7 days, how many days were you physically active for at least 60 minutes a day? (consider the time you spent in any kind of physical activity that increased your heart rate and made your breathing faster for some time). The response options were as follows: no day, 1 day, 2 days, 3 days, 4 days, 5 days, 6 days, and 7 days. Considering that physical activity recommendations for children and adolescents are 60 minutes of moderate and/or vigorous physical activity at least 5 days per week [17], participants who answered five or more days per week were classified as physically active and those who responded less than five days were classified as slightly physically active.

Fruit consumption was assessed in the last seven days preceding the interview by the following questions: how many times have you taken natural fruit juice? How often do you eat fruits? Each of the items had the following response options: I did not take fruit juice or did not eat fruits; 1 to 3 times; 4 to 6 times, once a day, 2 times daily, 3 times daily; 4 or more times daily. The responses to both questions were analyzed considering the recommendations of fruit consumption suggested by the Food Guide Pyramid [18], which suggests the consumption of 3 to 5 fruit servings daily. Thus, adolescents who consumed fruit and/or fruit juice three or more times daily were considered as “adequate consumption” and those who consumed less were considered as “inadequate consumption.”

The consumption of greens and vegetables was assessed in the last seven days prior to the interview through the questions: How many times did you eat green salad? How often do you eat potatoes? How many times did you eat carrots? How many times did you eat other vegetables? Each of the items had the following response options: I did not; 1 to 3 times; 4 to 6 times, once daily, 2 times daily, three times daily, and four or more times daily. The responses were analyzed considering the recommendations of fruits and vegetable consumption suggested by the Food Guide Pyramid [18], which suggests the consumption of 4-5 daily servings of greens and vegetables. Thus, adolescents who consumed greens and vegetables four or more times daily were considered as “adequate consumption” and those who consumed less were considered “inadequate consumption.”

Smoking was investigated by the question: during the past 30 days, on how many days did you smoke cigarettes? Participants who responded to have smoked at least once were considered a positive response (risk group) to smoking. Alcohol consumption was investigated by the question: during the past 30 days, on how many days you had at least one dose of alcohol (1 can of beer—340 mL or, 1 glass of wine—142 mL or, 1 shot spirits—42 mL)? Those who responded to have drunk at least once were considered a positive response (risk group) to the consumption of alcoholic beverages.

Sports team participation at the school or the outside school environment was analyzed by the following item: during the past 12 months, on how many team sports did you play? The response to this item was ranked as “≥2 sports team,” “1 sports team,” and “no sports team.”

### 2.7. Statistical Analysis

Descriptive and inferential statistics were applied. The linear trend and heterogeneity chi-square test was used to assess the association between excess TV time and independent variables. In the crude and adjusted association analysis, Wald test and binary logistic regression were used to estimate odds ratios (OR) and 95% confidence intervals (CI). Regression analysis followed a determination hierarchical model to identify factors related to excess TV time [19].

Statistical modeling followed the division into four groups of variables: (a) distal, which included demographic variables (age and skin color), (b) intermediate 1, which included socioeconomic variables (socioeconomic status and maternal and paternal schooling), (c) intermediate 2, which included health-related behaviors (physical activity, consumption of fruits, greens and vegetables, and smoking and alcohol use), and (d) proximal, which included sports team participation. All variables were adjusted. All variables were adjusted in the analyses, regardless of *P* value in the crude analysis.

In the adjusted analysis, a hierarchical approach was adopted, according to the backward procedure. Initially, the variables of the distal block were adjusted to the other factors of the same level. Then, the variables of the intermediate block 1 were controlled to the variables of the same level and to those of the distal level that remained in the model. Then, the variables of the intermediate block 2 were controlled to the variables of the same level and to those of intermediate 1 and distal level that remained in the model. Finally, in the analysis of variables of the proximal block, the adjustment was performed to the other factors of intermediate and distal levels that remained in the model. A *P* value below 0.20 in the Wald test was adopted as criterion for the permanence of the factor in the regression analyses [20]. Finally, factors with *P* < 0.05 were considered significantly associated with the outcome. There was an interaction between the variable gender and sedentary behavior (excessive television viewing); consequently analyses were stratified by gender using the Stata 11.0 software (StataCorp, College Station, Texas, USA).

## 3. Results

Of the 2105 volunteer participants 63% (*n* = 1327) were female. Most girls were less than 16 years of age, had lighter skinned black, intermediate economic level, and maternal and paternal schooling less than nine years. Nearly 90% of girls were slightly physically active, 79.4% consumed less than three daily servings of fruits, and 90.4% consumed less than four daily servings of greens and vegetables. The prevalence of smoking among girls was 6.0%, 37.8% drank alcoholic beverages, and 69.6% had not participated in any team sports in the 12 months preceding the survey (Table 1).

Most boys were aged less than 16 years, had lighter skinned black, intermediate economic level, and maternal and paternal schooling less than nine years. Almost 73.0% were slightly physically active, 78.0% consumed less than three daily fruit servings, and 90.2% consumed less than four daily servings of greens and vegetables. Smoking prevalence was 7.3%, 39.2% drank alcoholic beverages, and 37.9% had not participated in any team sports in the 12 months preceding the survey (Table 1).

The prevalence of excess TV time in girls was 70.9%. Girls of high economic level, whose paternal schooling was high, and with low consumption of greens and vegetables had higher prevalence of excess TV time (*P* < 0.05). For boys, the prevalence of excess TV time was 66.2%. Those with dark skinned black had higher prevalence of excess TV time (*P* < 0.05) (Table 2).

In the crude analysis (Table 3), girls of “high” and “intermediate” economic levels and with low consumption of greens and vegetables were more likely to have excess TV time. After adjustment, girls of “high” economic level and with low consumption of greens and vegetables were more likely to have excess TV time. Variable paternal schooling was on the threshold of association with excess TV time (*P* = 0.06), indicating that girls whose father had high educational level were more likely to have sedentary risk behavior compared to those whose father had low educational level.

In the crude analysis (Table 4), boys with dark skinned black were more likely to have excess TV time. After adjustment, students aged ≤16 years and with dark skinned black were more likely to have excess TV time.

## 4. Discussion

The main finding of this study was that the prevalence of excess TV time was very high, with slightly higher rates for girls. Correlated factors differed between girls and boys. In girls, high socioeconomic status and inadequate consumption of greens and vegetables were associated with excess TV time; in boys, being aged ≥16 years and dark skinned black were associated with this outcome.

Sedentary behaviors are distinct and independent from physical activity behaviors of various intensities >1.5 METs (i.e., light, moderate, and vigorous intensity) [1, 2]. Importantly, it is possible to meet physical activity recommendations and still engage in high amounts of sedentary behavior [3]. This paper focuses on excess TV time among young people, because in middle-income countries such as Brazil, the TV is cheaper than other electronics, such as computers and video games.

More girls than boys reported excess TV time, similar to results from adolescents in South Korea [21]. Research in the United States [22] examined different sedentary behaviors and reported that girls spent more time in sedentary activities than boys when the activity was sitting listening to music and talking on the phone; conversely, when the activity was to play video games, boys spent more time sitting than girls. In relation to time spent watching television, there were no differences between sexes [22]. As in the United States, adolescents aged 13 to 18 years from China also showed no differences between sexes when analyzing time spent watching TV [23]. Salmon et al. [8] summarized research from around the world and showed that 17–94% of adolescents of similar age failed to meet screnn time guidelines, but no sex breakdown was provided.

This study revealed that girls of high socioeconomic status were more likely to have excess TV time than those of low socioeconomic status. In a cohort study in Pelotas, Brazil [24], as in a cross-sectional study in the United States [25], adolescents with high socioeconomic status were more likely to have excess sedentary behavior. One possible explanation for this may be the fact that wealthier families have greater purchasing power and acquire more electronic equipment such as computers, video games, and televisions than poorer families, and this increased purchasing power favors increasingly sedentary activities, low physical activity levels, and consequently increased sedentary risk behavior [7].

This study also found low consumption of greens and vegetables was associated with excess TV time in girls. Similar results were reported in young people from Norway [26] and Pakistan [27]. The association between TV time and other sedentary risk behaviors and low consumption of greens and vegetables can be one of the environmental factors responsible for obesity [27]. Children and adolescents who spend excess time watching TV tend to feed improperly and, as a result, show greater body adiposity and metabolic disorders [21, 28]. In this sense, there is need for guidance in the school environment in order to reduce sedentary behavior through the practice of physical and sport activities while encouraging increasingly healthy eating habits among young people.

Older boys in this study were more likely to have excess TV time than younger ones. In research conducted in California, United States, no association was found between TV time and age [25]. In South Korea, young boys and girls (<16 years old) spent more time during the week watching TV than older ones (≥16 years) [21]. The lack of consensus between studies reinforces what has been found in a recent literature review on the subject, which pointed out that there is insufficient evidence regarding the factors associated with sedentary behavior in children and adolescents [8, 29]. Furthermore, there is limited evidence on the relationships among sedentary behaviors (of different types), correlates of sedentary behaviors, and subsequent health consequences.

In boys, dark skinned black was a factor associated with excess TV time. This finding is consistent with previous studies [25, 30] and inconsistent with a cohort study carried out in Brazil [24]. Further research is required to determine and resolve racial disparities in this and other lifestyle behaviors.

This study found no association between physical activity or sports team participation and excess TV time. This reinforces evidence that sedentary behaviors and physical activity behaviors often emerge independently and each needs to be addressed for public health purposes [2]. Other studies have found results similar to those of the present study in this regard [2, 6, 8, 31].

This study has some limitations, including the following. The analyses are based on cross-sectional data; therefore, a cause and effect relationship between independent variables and sedentary behavior cannot be drawn. The focus of the study was to analyze individual variables, which limit the inferences to the context of the person. Another limitation of this study was the investigation of only one sedentary behavior (time spent watching TV) among different existing sedentary behaviors (use of computer, video game, time sitting talking, reading, and talking on the phone). Data were not collected on whether adolescents had access to TV in their bedroom; this may be an important variable to collect in future research.

A major strength of this study is the use of data from an understudied population; adolescents primarily not from high social levels (almost 80% were from middle or lower SES status) enrolled in public high schools in Northeastern Brazil. The representative sample of this region of Brazil is another strong point of the study.

## 5. Conclusion

It could be concluded that adolescents from Aracaju, Brazil, have high prevalence of excess TV time, which may increase risk for future chronic diseases in this population. More girls have excess TV time than boys though in both sexes the prevalence is very high. Factors related to excess TV time differ according to sex, suggesting that intervention strategies may need to be tailored differently for girls and boys.

## Figures and Tables

**Table 1:** Sample distribution and descriptive characteristics according to sex.

Variables	Female		Male	
	*n*	% (95% CI)	*n*	% (95% CI)
Total	**1327**	**100**	**778**	**100**
Age (years)				
≤16	762	57.6 (54.9, 60.2)	440	56.8 (53.2, 60.2)
17-18	561	42.4 (39.7, 45.0)	335	43.2 (39.7, 46.7)
Skin color				
White	173	14.9 (12.9, 17.0)	108	16.0 (13.2, 18.8)
Lighter skinned black	725	62.8 (59.9, 65.5)	401	59.5 (55.7, 63.2)
Dark skinned black	258	22.3 (19.9, 24.7)	165	24.5 (21.2, 27.7)
Socioeconomic status				
High	277	21.1 (18.8, 23.3)	217	28.2 (25.0, 31.4)
Intermediate	857	65.3 (62.6, 67.8)	470	61.3 (57.8, 64.7)
Low	179	13.6 (11.7, 15.4)	80	10.5 (8.2, 12.5)
Maternal schooling				
>8 years	458	36.0 (33.3, 38.5)	309	41.7 (38.1, 45.2)
≤8 years	816	64.0 (61.4, 66.6)	432	58.3 (54.7, 61.8)
Paternal schooling				
>8 years	452	37.3 (34.5, 39.9)	298	42.0 (38.3, 45.3)
≤8 years	761	62.7 (60.0, 65.4)	411	58.0 (54.3, 61.3)
Physical activity				
Active	166	12.6 (10.7, 14.3)	210	27.2 (24.0, 30.3)
Little active	1156	87.4 (85.6, 89.2)	563	72.8 (69.6, 75.9)
Fruit consumption				
≥3 portions/day	273	20.6 (18.3, 22.7)	170	22.0 (19.0, 24.8)
<3 portions/day	1054	79.4 (77.2, 81.6)	604	78.0 (75.1, 80.9)
Vegetables consumption				
≥4 portions/day	127	9.6 (7.9, 11.1)	76	9.8 (7.6, 11.8)
<4 portions/day	1200	90.4 (88.8, 92.0)	702	90.2 (88.1, 92.3)
Smoking				
No	1241	94.0 (92.7, 95.2)	721	92.7 (90.8, 94.5)
Yes	79	6.0 (4.7, 7.2)	57	7.3 (5.5, 9.1)
Alcohol use				
No	823	62.2 (59.5, 64.8)	470	60.8 (57.3, 64.2)
Yes	500	37.8 (35.1, 40.4)	303	39.2 (35.7, 42.6)
Sports team participation				
≥2 Sports team	104	7.9 (6.4, 9.3)	257	33.3 (29.9, 36.6)
1 Sports team	295	22.5 (20.1, 24.7)	222	28.8 (25.5, 31.9)
No Sports team	915	69.6 (67.1, 72.1)	292	37.9 (34.4, 41.3)

CI: confidence interval.

**Table 2:** Prevalence of excess television time (≥2 hours/day) according to independent variables.

Variables	Female		Male	
	*n*	% (95% CI)	*n*	% (95% CI)
Total	**941**	**70.9 (68.4, 73.3)**	**515**	**66.2 (62.8, 69.5)**
Age (years)		*P* = 0.09*		*P* = 0.08*
≤16	554	72.7 (69.5, 75.8)	302	68.6 (64.2, 72.9)
17-18	384	68.5 (64.5, 72.3)	210	62.7 (57.4, 67.8)
Skin color		*P* = 0.17^†^		*P* = 0.05^†^
White	128	73.9 (67.3, 80.5)	68	62.9 (53.7, 72.2)
Lighter skinned black	499	68.8 (65.4, 72.2)	260	64.8 (60.1, 69.5)
Dark skinned black	191	74.0 (68.6, 79.4)	123	74.6 (67.8, 81.2)
Socioeconomic status		*P* = 0.03*		*P* = 0.07*
High	204	73.6 (68.4, 78.8)	131	60.3 (53.8, 66.9)
Intermediate	614	71.6 (68.6, 74.6)	325	69.2 (64.9, 73.3)
Low	114	63.6 (56.5, 70.8)	52	65.0 (54.3, 75.6)
Maternal schooling		*P* = 0.97^†^		*P* = 0.48^†^
>8 years	324	70.7 (66.5, 74.9)	210	67.9 (62.7, 73.1)
≤8 years	578	70.8 (67.7, 73.9)	283	65.5 (61.0, 70.0)
Paternal schooling		*P* = 0.05*		*P* = 0.95*
>8 years	334	73.8 (69.8, 77.9)	198	66.4 (61.0, 71.8)
≤8 years	523	68.7 (65.4, 72.0)	274	66.6 (62.1, 71.2)
Physical activity		*P* = 0.16^†^		*P* = 0.22^†^
Active	110	66.2 (58.9, 73.5)	146	69.5 (63.2, 75.8)
Little active	827	71.5 (68.9, 74.1)	365	64.8 (60.8, 68.7)
Fruit consumption		*P* = 0.69*		*P* = 0.88*
≥3 portions/day	191	69.9 (64.4, 75.4)	113	66.4 (59.3, 73.6)
<3 portions/day	750	71.2 (68.4, 73.8)	398	65.8 (62.1, 69.6)
Vegetables consumption		*P* = 0.03*		*P* = 0.27*
≥4 portions/day	80	62.9 (54.4, 71.5)	46	60.5 (49.2, 71.7)
<4 portions/day	861	71.7 (69.1, 74.3)	469	66.8 (63.3, 70.3)
Smoking		*P* = 0.13^†^		*P* = 0.93^†^
No	876	70.5 (68.0, 73.1)	477	66.1 (62.6, 69.6)
Yes	62	78.4 (69.2, 87.7)	38	66.6 (54.0, 79.2)
Alcohol use		*P* = 0.82^†^		*P* = 0.60^†^
No	583	70.8 (67.7, 73.9)	308	65.5 (61.2, 69.8)
Yes	357	71.4 (67.4, 75.3)	204	67.3 (62.0, 72.6)
Sports team participation		*P* = 0.99*		*P* = 0.37*
≥2 Sports team	74	71.1 (62.3, 80.0)	175	68.0 (62.3, 73.8)
1 Sports team	209	70.8 (65.6, 76.0)	152	68.4 (62.3, 74.6)
No Sports team	649	70.9 (67.9, 73.8)	185	63.3 (57.7, 68.9)

CI: confidence interval; *linear trend chi-square test; ^†^heterogeneity chi-square test.

**Table 3:** Estimated odds ratios and 95% confidence intervals in the association between excess television time and independent variables in girls.

Variables	Crude analysis		*P*	Adjusted analysis*		*P*
	OR	(95% CI)		OR	(95% CI)	
Age (years)^1^						
≤16	1.0		0.09	1.0		0.11
17-18	0.8	(0.6, 1.0)		0.8	(0.6, 1.1)	
Skin color^1^						
White	1.0		0.24	1.0		0.28
Lighter skinned black	0.8	(0.5, 1.1)		0.7	(0.5, 1.2)	
Dark skinned black	1.1	(0.6, 1.6)		1.0	(0.5, 1.8)	
Socioeconomic status^2^						
High	1.6	(1.1, 2.3)	0.05	1.6	(1.1, 2.2)	0.05
Intermediate	1.4	(1.1, 2.0)		1.4	(1.0, 1.9)	
Low	1.0			1.0		
Maternal schooling^2^						
>8 years	1.0		0.97	1.0		0.32
≤8 years	1.1	(0.8, 1.2)		1.2	(0.8, 1.5)	
Paternal schooling^2^						
>8 years	1.0		0.06	1.0		0.06
≤8 years	0.7	(0.5, 1.0)		0.7	(0.6, 1.0)	
Physical activity^3^						
Active	1.0		0.16	1.0		0.24
Little active	1.3	(0.9, 1.8)		1.2	(0.8, 1.7)	
Fruit consumption^3^						
≥3 portions/day	1.0		0.69	1.0		0.99
<3 portions/day	1.1	(0.8, 1.4)		1.0	(0.7, 1.3)	
Vegetables consumption^3^						
≥4 portions/day	1.0		0.04	1.0		0.02
<4 portions/day	1.5	(1.1, 2.1)		1.6	(1.1, 2.3)	
Smoking^3^						
No	1.0		0.13	1.0		0.08
Yes	1.5	(0.8, 2.6)		1.7	(0.9, 3.0)	
Alcohol use^3^						
No	1.0		0.82	1.0		0.77
Yes	1.1	(0.8, 1.3)		0.9	(0.7, 1.3)	
Sports team participation^3^						
≥2 Sports team	1.0		0.99	1.0		0.86
1 Sports team	0.9	(0.6, 1.6)		0.9	(0.5, 1.6)	
No Sports team	0.9	(0.6, 1.5)		0.9	(0.6, 1.4)	

OR: odds ratio; CI: confidence interval; *adjusted Analysis: all variables were included in the adjusted analysis regardless of *P* value in the crude analysis. Adjusted model variables were retained with *P* value ≤ 0.20; superscript numbers (1, 2, 3, and 4) represent the input of variables in the hierarchic model for the adjusted analysis.

**Table 4:** Estimated odds ratios and 95% confidence intervals in the association between excess television time and independent variables in boys.

Variables	Crude analysis		*P *	Adjusted analysis*		*P *
	OR	(95% CI)		OR	(95% CI)	
Age (years)^1^						
≤16	1.0		0.08	1.0		0.03
17-18	0.7	(0.5, 1.0)		0.7	(0.5, 0.9)	
Skin color^1^						
White	1.0		0.05	1.0		0.04
Lighter skinned black	1.1	(0.6, 1.7)		1.1	(0.7, 1.7)	
Dark skinned black	1.7	(1.1, 2.9)		1.8	(1.1, 3.1)	
Socioeconomic status^2^						
High	0.8	(0.4, 1.3)	0.07	0.9	(0.5, 1.4)	0.08
Intermediate	1.2	(0.7, 1.9)		1.2	(0.8, 2.0)	
Low	1.0			1.0		
Maternal schooling^2^						
>8 years	1.0		0.48	1.0		0.26
≤8 years	0.9	(0.6, 1.2)		0.8	(0.5, 1.1)	
Paternal schooling^2^						
>8 years	1.0		0.95	1.0		0.96
≤8 years	1.1	(0.7, 1.3)		1.0	(0.7, 1.4)	
Physical activity^3^						
Active	1.0		0.22	1.0		0.29
Little active	0.9	(0.5, 1.1)		0.8	(0.5, 1.1)	
Fruit consumption^3^						
≥3 portions/day	1.0		0.88	1.0		0.46
<3 portions/day	0.9	(0.7, 1.4)		0.9	(0.6, 1.3)	
Vegetables consumption^3^						
≥4 portions/day	1.0		0.27	1.0		0.39
<4 portions/day	1.3	(0.8, 2.1)		1.3	(0.7, 2.0)	
Smoking^3^						
No	1.0		0.93	1.0		0.94
Yes	1.1	(0.6, 1.8)		1.1	(0.5, 1.9)	
Alcohol use^3^						
No	1.0		0.60	1.0		0.19
Yes	1.1	(0.7, 1.4)		1.2	(0.9, 1.7)	
Sports team participation^3^						
≥2 Sports team	1.0		0.37	1.0		0.33
1 Sports team	1.0	(0.6, 1.4)		0.9	(0.6, 1.4)	
No Sports team	0.8	(0.5, 1.2)		0.8	(0.5, 1.1)	

OR: odds ratio; CI: confidence interval; *adjusted analysis: All variables were included in the adjusted analysis regardless of *P* value in the crude analysis. Model variables were retained with *P* value ≤ 0.20; superscript numbers (1, 2, 3, and 4) represent input of variables in the hierarchic model for the adjusted analysis.
